# Obstetrics

**Published:** 1860-09

**Authors:** 


### OBSTETRICS.

ON IODINE INJECTIONS IN OVARIAN CYSTS. By *Professor Scuh.*—The primary action of iodine injections on ovarian cysts is remarkably variable; and that not only according to the quantity and dilution of the tincture, the amount of the still remaining contents of the cysts, and the peculiarities of the individual, but also according to the condition of the cyst itself as regards its thickness, solidity, its connections with surrounding parts, its surface, and the abundance or scarcity of the supply of vessels—leading to the greater or less stimulation of the cyst, and hastening or delaying the passage of the injected material into the blood, as well as its influence on the nervous system. The peculiarities of the walls of the cyst due to their permeability, and their capability of exosmose and endosmose, cannot be determined beforehand with any exactitude, not only in different patients, but even in the same patient, in case of repetition of the injections—changes being determined in the texture of the sac which cannot be appreciated. A certain quantity of the tincture, which on the first occasion scarcely exerted any local or general influence whatever, may on a repetition of the injection give rise to the most violent and dangerous symptoms. This is the more extraordinary because the same disparity of effect is not observed in other affections in which iodine injections are employed, *e.g.* ascites, abscess, thyroid cysts, enlarged bursæ, etc.

The author has studied the primary effects of injections on fifteen occasions. Sometimes there is no pain or tenderness on pressure; or if the latter exists to some extent, it only lasts for a day or two, the iodine freely passing into the urine from the commencement. Sometimes severe pain is produced at the time of the injection, which persists with exacerbation, or it only first comes on some time after the operation. At the same time a great change in the general condition is noticed—restlessness, vomiting, sleeplessness, faintness, and rapidity of pulse being among the symptoms. In some cases the pulse remains unchanged, but great alteration has taken place in the countenance. In other instances the pulse is very small and feeble, as well as rapid, the extremities are cold, and consciousness is temporarily lost—alarming symptoms that may continue for several hours or a day, and then gradually cease. Not only is iodine found in the urine a few minutes after injection, but likewise in the saliva and in the vomited matters; this iodine reaction being exhibited for from four to twelve days, although the other symptoms have usually terminated earlier. In some cases the primary influence seems to be expended on the cyst and its vicinity, since great pain and tenderness arise, to be followed by shivering and heat, indicating either suppurative inflammation of the cyst, or the development of a dangerous peritonitis. There can be no doubt that the symptoms of poisoning above mentioned arise chiefly from the rapid passage of the iodine into the blood; but although the nervous system may be principally affected, through this, its becoming so so rapidly in some cases also indicates a primary action of the iodine upon it.

The *indication for the iodine injection* is the existence of a unilocular globular cyst which has not reached too great a size, having but thin walls, presenting an equal resistance at all points, and containing thin, serous fluid. In order the better to judge of these points, a preliminary puncture of the cyst should always be made, discharging the contents as far as possible. The manner in which this preliminary puncture is borne—*i.e.* with respect to the amount of irritation produced—will give some idea as to the quantity and concentration of the fluid which is to be hereafter injected. This injection should be proceeded with as soon as the fluid has collected again in sufficient quantity for a puncture to be made without risk of injury to the intestines, it being by no means desirable to wait until the tumor has reacquired its former volume. When after the preliminary puncture a large mass is still left behind, we may conclude either that the walls of the cyst are thick and vascular, that there are multiple cysts, that there are villous or other pediculated growths from the inner wall, or that the cyst is interwoven with fibrous or other parenchymatous structure. These circumstances diminish greatly the chance of success, or they prohibit the performance of the injection. In some rare cases, indeed, in which two or three cysts have existed, the injection of the largest of these has sufficed for a cure; the iodine, through the operation of endosmose or exosmose, exerting its influence upon the smaller cysts; or the smaller cysts having become perforated through the larger, the remedy thus gains access to all. But upon such exceptional instances the surgeon cannot count. In very large cysts, by which great traction of the viscera, dyspnœa, etc., have been induced, and in cysts exhibiting irregularities of surface and indurations, which may arise from the aggregation of numerous cysts, or from the presence of fibrous, carcinomatous, or other degenerated masses, the idea of the injection should be entirely abandoned. It would only lead to a more rapid growth, or give rise to suppurative inflammation. When the puncture gives issue to a thick, almost gelatinous fluid, this indicates a condition of the walls of the cysts not easily influenced by iodine. It is very rare for such fluid to become thinner and more serous on subsequent punctures.

The fluid to be injected should amount to from two to six ounces, consisting of tincture of iodine diluted by from one to eight parts of water, adding a scruple of iodide of potassium. As the extent of its stimulating power cannot be always foreseen, it is best, especially on the first occasion, and when the cyst has been nearly emptied, not to employ it too concentrated. The desirableness of preventing access of air during the injection is obvious: and the canula and tube affixed to the syringe are best made of platinum, this being the metal upon which iodine exerts least action. The fluid is not to be allowed to run out again; but should very severe pains follow immediately after the injection, some water should be thrown in, in order to effect dilution. The stimulation of the cyst by the iodine usually leads to an inflammation which is limited to the walls of the cyst. The serous exudation is increased, and the tumor in a few days reacquires the size it had prior to the operation. After then the size again gradually diminishes, and it is a very favorable sign when with such diminution in size an increase of resistance or an actual induration is perceived. This latter condition is due to coagulation of albuminous matters induced by the iodine, and these are often so thick that a repetition of the puncture at this period gives issue to no fluid, or only a very small quantity. After weeks or even months further changes take place, in virtue of which the coagula disappear, and the contents of the sac again become serous. As long as any diminution in the size of the tumor is observed, however slow in progress this may be, no repetition of the operation should take place; nor should such repetition be put into force as long as any considerable tenderness on pressure remains. It is indicated when the inflammatory condition has been quite transitory, and when the enlargement takes on an increase or remains completely stationary for longer than six weeks. In some cases the repetition may be necessary from two to six times. When from the entrance of air, or other cause, foul suppuration, with extrication of gas, is engendered, and there is tenderness on pressure, fever and loss of strength, a simple puncture should be made, and a catheter left in or the aperture enlarged. In all such cases a most careful cleansing of the cavity by means of repeated injections of water or decoction of bark should be effected. It has been said that by repeated iodine injections the contents of a cyst may be converted from a purulent to a serous fluid; but in this statement the author puts no trust, a similar result never being produced by injection of abscesses.

The statistics of the results of iodine injection are given very differently by different authors. This seems to have arisen more from the mode in which the cases were chosen, and the degree of exactitude with which they were reported, than from trifling differences in the operative procedure. The author can only speak personally respecting six cases for which fifteen injections were employed. In only one of these did complete recovery take place, and that after a second injection. It was the only one of the six which united all the conditions necessary to secure a favorable issue. In a second case the cyst was bilocular, and a slight diminution of its size only resulted from five injections. The others were multilocular cysts, or the cysts contained a thick, gelatinous fluid—constituting cases which were, according to the author's present conviction, unsuited for iodine injection. In two of them no essential change was produced, and in the others the end of the patient seemed to have been hastened, partly through the speedy repetition of the operation, and partly through suppurative inflammation and peritonitis being induced.—*Zeitschrift der Aerzte zu Wein*, 1859, No. 48.—(*Medical Times and Gazette*, June 16, 1860.)

